# Supplementary figures and images for: Impact of benzodiazepine use on the risk of occupational accidents
Source: PLoS One. 2024 Apr 16;19(4):e0302205. doi: 10.1371/journal.pone.0302205 (PMC11020385; doi:10.1371/journal.pone.0302205)

**Figure S1. Frequency of people by age in 2017.**

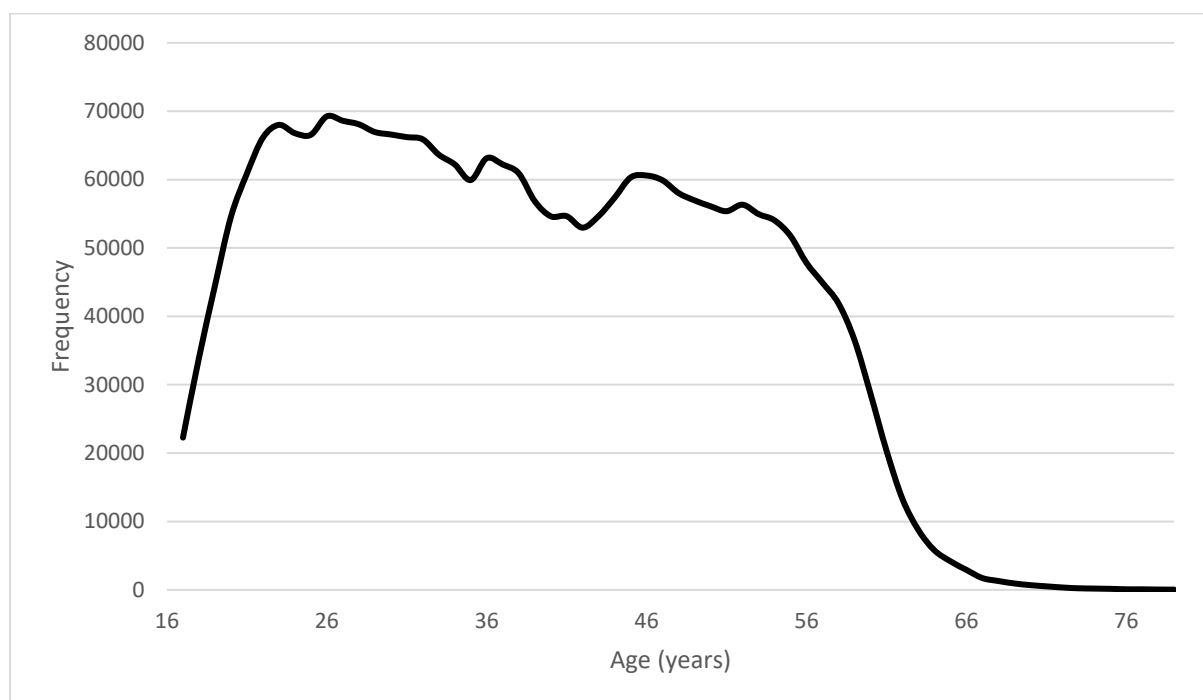

Field: Population having had at least one WA from 2017 to 2019 (N = 2,544,237).

Supplement: S1 Fig — Field: Population having had at least one WA from 2017 to 2019 (N = 2,544,237). (PDF) [file pone.0302205.s001.pdf]
