# Supplementary material for: Impact of benzodiazepine use on the risk of occupational accidents
Source: PLoS One. 2024 Apr 16;19(4):e0302205. doi: 10.1371/journal.pone.0302205 (PMC11020385; doi:10.1371/journal.pone.0302205)
Supplement: S1 Table — Field: Population having had at least one WA from 2017 to 2019 (N = 2,544,237). Note: * p < 0.05, ** p < 0.01, *** p < 0.001. Standard errors in parentheses. Interpretation: For men, BZD overuse (compared to no BZD use, calculated for months t-4 to t-1) is associated with a 0.097 pp increase in WA probability at month t. (PDF) [file pone.0302205.s002.pdf]

S1 Table. Regressions of WA risk by sex.

	**Men**	**Women**
BZDs (ref. no use)		
Overuse	0.00097*	0.00053
	(0.00048)	(0.00042)
Recent use	-0.00147***	-0.00206***
	(0.00024)	(0.00021)
Past use	0.00138***	0.00101***
	(0.00018)	(0.00016)
Chronic conditions		
Psychiatric	-0.0179***	-0.02594***
	(0.00085)	(0.00078)
Other diseases	-0.00739***	-0.01535***
	(0.00041)	(0.00047)
Drugs reimbursed		
No other psycholeptics	-0.0024***	-0.00255***
	(0.00051)	(0.00047)
Other psycholeptics (log(€))	-0.00079***	-0.0009***
	(0.00023)	(0.00023)
No antidepressants	0.00204**	0.00255***
	(0.00069)	(0.00054)
Antidepressants (log(€))	0.00096***	0.00154***
	(0.00026)	(0.00021)
No other drugs	0.0045***	0.00068***
	(0.00014)	(0.00016)
Other drugs (log(€))	0.00062***	0.00045***
	(0.00004)	(0.00004)
Doctor consultations		
GP	-0.00258***	-0.0028***
	(0.00002)	(0.00002)
Psychiatrist	0.00008	-0.00015**
	(0.00007)	(0.00005)
Other specialists	-0.00204***	-0.00183***
	(0.00005)	(0.00004)
Absence from work		
Compensated days off work	-0.00041***	-0.00035***
	(0)	(0)
Hospitalization days	-0.0002***	-0.0002***
	(0.00001)	(0.00001)
Fixed effects		
Individual	Yes	Yes
Time	Yes	Yes
R^2	0.015205	0.013566
**Observations**	**1,480,432**	**1,063,805**

Field: Population having had at least one WA from 2017 to 2019 (N = 2,544,237). Note: * $p < 0.05$, ** $p < 0.01$, *** $p < 0.001$. Standard errors in parentheses. Interpretation: For men, BZD overuse (compared to no BZD use, calculated for months t-4 to t-1) is associated with a 0.097 pp increase in WA probability at month t.
