# Supplementary material for: Impact of benzodiazepine use on the risk of occupational accidents
Source: PLoS One. 2024 Apr 16;19(4):e0302205. doi: 10.1371/journal.pone.0302205 (PMC11020385; doi:10.1371/journal.pone.0302205)
Supplement: S2 Table — Field: Population having had at least one WA from 2017 to 2019 (N = 2,544,237). Note: * p < 0.05, ** p < 0.01, *** p < 0.001. Standard errors in parentheses. Interpretation: For people under 30 years old, BZD overuse (compared to no BZD use, calculated for months t-4 to t-1) is not significantly (at a 5% threshold) associated with WA probability at month t. (PDF) [file pone.0302205.s003.pdf]

S2 Table. Regressions of WA risk by age.

	< 30 years	30-44 years	45-59 years	> 59 years
BZDs (ref. no use)				
Overuse	0.00154	0.00211***	0.00053	-0.00304*
	(0.00109)	(0.00054)	(0.00044)	(0.00138)
Recent use	-0.0027***	-0.002***	-0.00122***	-0.00153
	(0.00038)	(0.00026)	(0.00024)	(0.00079)
Past use	0.00135***	0.00118***	0.0013***	-0.00027
	(0.00028)	(0.0002)	(0.00019)	(0.00065)
Chronic conditions				
Psychiatric	-0.01641***	-0.02042***	-0.02589***	-0.02785***
	(0.00125)	(0.00094)	(0.00093)	(0.00368)
Other diseases	-0.02574***	-0.00916***	-0.00613***	-0.00847***
	(0.00082)	(0.0006)	(0.00042)	(0.00118)
Drugs reimbursed				
No other psycholeptics	-0.00222**	-0.00175**	-0.00373***	-0.00732***
	(0.00067)	(0.00055)	(0.00059)	(0.00213)
Other psycholeptics	-0.00025	-0.0008**	-0.00163***	-0.00276*
(log(€))	(0.00032)	(0.00026)	(0.00028)	(0.00107)
No antidepressants	0.00211*	0.00232***	0.00191**	0.00443
	(0.00107)	(0.00069)	(0.00065)	(0.00227)
Antidepressants (log(€))	0.00175***	0.0014***	0.00095***	0.00202*
	(0.00043)	(0.00027)	(0.00025)	(0.00086)
No other drugs	0.00245***	0.00412***	0.00444***	0.00401***
	(0.00018)	(0.00017)	(0.0002)	(0.00074)
Other drugs (log(€))	0.00093***	0.00058***	0.00035***	0.00006
	(0.00006)	(0.00005)	(0.00006)	(0.0002)
Doctor consultations				
GP	-0.00231***	-0.00273***	-0.00307***	-0.0029***
	(0.00003)	(0.00002)	(0.00003)	(0.00008)
Psychiatrist	0.00013	-0.00005	-0.00012	-0.00029
	(0.0001)	(0.00007)	(0.00007)	(0.00025)
Other specialists	-0.00206***	-0.00189***	-0.00178***	-0.00175***
	(0.00006)	(0.00005)	(0.00005)	(0.00016)
Absence from work				
Compensated days off	-0.00042***	-0.00038***	-0.00035***	-0.00028***
work	(0)	(0)	(0)	(0)
Hospitalization days	-0.00024***	-0.0002***	-0.00018***	-0.00023***
	(0.00002)	(0.00001)	(0.00001)	(0.00003)
Fixed effects				
Individual	Yes	Yes	Yes	Yes
Time	Yes	Yes	Yes	Yes
R^2	0.014516	0.014707	0.014425	0.016744
**Observations**	**823,029**	**895,579**	**763,944**	**61,685**

Field: Population having had at least one WA from 2017 to 2019 (N = 2,544,237). Note: * $p < 0.05$, ** $p < 0.01$, *** $p < 0.001$. Standard errors in parentheses. Interpretation: For people under 30 years old, BZD overuse (compared to no BZD use, calculated for months t-4 to t-1) is not significantly (at a 5% threshold) associated with WA probability at month t.
