# Supplementary material for: Impact of benzodiazepine use on the risk of occupational accidents
Source: PLoS One. 2024 Apr 16;19(4):e0302205. doi: 10.1371/journal.pone.0302205 (PMC11020385; doi:10.1371/journal.pone.0302205)
Supplement: S3 Table — Field: Population having had a single WA from 2017 to 2019 (N = 2,170,144). Note: * p < 0.05, ** p < 0.01, *** p < 0.001. Standard errors in parentheses. Interpretation: For people whose WA did not lead to a work stoppage, BZD overuse (compared to no BZD use, calculated for months t-4 to t-1) is not significantly (at a 5% threshold) associated with WA probability at month t. (PDF) [file pone.0302205.s004.pdf]

S3 Table. Regressions of WA risk by duration of work stoppage following the WA.

	**0 day**	**1-7 days**	**8-26 days**	**> 26 days**
BZDs (ref. no use)				
Overuse	0.00016<br>(0.00049)	-0.0005<br>(0.00092)	0.00172*<br>(0.00075)	0.00045<br>(0.00059)
Recent use	-0.0005*<br>(0.00025)	-0.00174***<br>(0.00045)	-0.00167***<br>(0.00036)	-0.00133***<br>(0.0003)
Past use	0.00044*<br>(0.00019)	0.00042<br>(0.00035)	0.00068*<br>(0.00028)	0.00198***<br>(0.00024)
Chronic conditions				
Psychiatric	-0.00879***<br>(0.00092)	-0.01768***<br>(0.00171)	-0.0214***<br>(0.00138)	-0.03282***<br>(0.00103)
Other diseases	-0.01766***<br>(0.00045)	-0.0074***<br>(0.00092)	-0.00445***<br>(0.00074)	-0.01129***<br>(0.00058)
Drugs reimbursed				
No other psycholeptics	-0.0014**<br>(0.00052)	0.0005<br>(0.00094)	-0.00238**<br>(0.00078)	-0.00498***<br>(0.00069)
Other psycholeptics (log(€))	-0.00067**<br>(0.00025)	-0.00008<br>(0.00044)	-0.00065<br>(0.00037)	-0.00188***<br>(0.00032)
No antidepressants	0.00143*<br>(0.00066)	0.00269*<br>(0.00123)	0.00092<br>(0.00101)	0.00162*<br>(0.0008)
Antidepressants (log(€))	0.00077**<br>(0.00025)	0.00167***<br>(0.00047)	0.00162***<br>(0.00039)	-0.00008<br>(0.00031)
No other drugs	0.00383***<br>(0.00015)	0.00537***<br>(0.00027)	0.00361***<br>(0.00023)	-0.00082***<br>(0.00022)
Other drugs (log(€))	0.00063***<br>(0.00005)	0.00118***<br>(0.00009)	0.00089***<br>(0.00007)	-0.00036***<br>(0.00006)
Doctor consultations				
GP	-0.00175***<br>(0.00002)	-0.00311***<br>(0.00005)	-0.0033***<br>(0.00003)	-0.00173***<br>(0.00003)
Psychiatrist	-0.00018**<br>(0.00006)	0.00002<br>(0.00012)	0.00011<br>(0.0001)	-0.00025**<br>(0.00008)
Other specialists	-0.00149***<br>(0.00005)	-0.00155***<br>(0.0001)	-0.00172***<br>(0.00008)	-0.00253***<br>(0.00006)
Absence from work				
Compensated days off work	-0.00005***<br>(0)	-0.0003***<br>(0)	-0.00039***<br>(0)	-0.00043***<br>(0)
Hospitalization days	-0.00019***<br>(0.00001)	-0.00027***<br>(0.00002)	-0.00022***<br>(0.00002)	-0.0002***<br>(0.00001)
Fixed effects				
Individual	Yes	Yes	Yes	Yes
Time	Yes	Yes	Yes	Yes
R^2	0.011222	0.002104	0.003265	0.010045
**Observations**	**773,685**	**351,821**	**537,581**	**507,057**

Field: Population having had a single WA from 2017 to 2019 (N = 2,170,144). Note: * $p < 0.05$, ** $p < 0.01$, *** $p < 0.001$. Standard errors in parentheses. Interpretation: For people whose WA did not lead to a work stoppage, BZD overuse (compared to no BZD use, calculated for months t-4 to t-1) is not significantly (at a 5% threshold) associated with WA probability at month t.
