# Supplementary material for: Impact of benzodiazepine use on the risk of occupational accidents
Source: PLoS One. 2024 Apr 16;19(4):e0302205. doi: 10.1371/journal.pone.0302205 (PMC11020385; doi:10.1371/journal.pone.0302205)
Supplement: S4 Table — Field: Population having had one single WA from 2017 to 2019 (N = 2,170,144). Note: * p < 0.05, ** p < 0.01, *** p < 0.001. Standard errors in parentheses. Interpretation: BZD overuse (compared to no BZD use, calculated for months t-4 to t-1) is not significantly (at a 5% threshold) associated with WA probability at month t. (PDF) [file pone.0302205.s005.pdf]

S4 Table. Regression of WA risk for the population with one single WA throughout the study period.

	Marginal effect (SE)
BZDs (ref. no use)	
Overuse	0.00056 (0.00032)
Recent use	-0.00114*** (0.00016)
Past use	0.00104*** (0.00012)
Chronic conditions	
Psychiatric	-0.02078*** (0.00058)
Other diseases	-0.01126*** (0.00031)
Drugs reimbursed	
No other psycholeptics	-0.00238*** (0.00035)
Other psycholeptics (log(€))	-0.00088*** (0.00016)
No antidepressants	0.00181*** (0.00043)
Antidepressants (log(€))	0.00097*** (0.00016)
No other drugs	0.00311*** (0.0001)
Other drugs (log(€))	0.00054*** (0.00003)
Doctor consultations	
GP	-0.00233*** (0.00002)
Psychiatrist	-0.00006 (0.00004)
Other specialists	-0.00179*** (0.00003)
Absence from work	
Compensated days off work	-0.00032*** (0)
Hospitalization days	-0.00019*** (0.00001)
Fixed effects	
Individual	Yes
Time	Yes
R^2	0.007353
**Observations**	**2,170,144**

Field: Population having had one single WA from 2017 to 2019 (N = 2,170,144). Note: * $p < 0.05$, ** $p < 0.01$, *** $p < 0.001$. Standard errors in parentheses. Interpretation: BZD overuse (compared to no BZD use, calculated for months t-4 to t-1) is not significantly (at a 5% threshold) associated with WA probability at month t.
