# Supplementary material for: Impact of benzodiazepine use on the risk of occupational accidents
Source: PLoS One. 2024 Apr 16;19(4):e0302205. doi: 10.1371/journal.pone.0302205 (PMC11020385; doi:10.1371/journal.pone.0302205)
Supplement: S5 Table — Field: Population having had a single WA from 2017 to 2019, resulting in at least a 4-day work stoppage, and for whom the information is available (N = 411,519). Note: * p < 0.05, ** p < 0.01, *** p < 0.001. Standard errors in parentheses. Interpretation: For people with a permanent contract at the time of WA, BZD overuse (compared to no BZD use, calculated for months t-4 to t-1) is associated with a 0.302 pp increase in WA probability at month t. (PDF) [file pone.0302205.s006.pdf]

S5 Table. Regressions of WA risk by type of employment contract.

	**Permanent contract**	**Fixed-term contract**
BZDs (ref. no use)		
Overuse	0.00302***	0.00534***
	(0.00062)	(0.00121)
Recent use	-0.00077*	0.00059
	(0.00032)	(0.00064)
Past use	0.00151***	0.00212***
	(0.00025)	(0.0005)
Chronic conditions		
Psychiatric	-0.00551***	0.00119
	(0.00108)	(0.00216)
Other diseases	0.01012***	0.00916***
	(0.00061)	(0.00128)
Drugs reimbursed		
No other psycholeptics	-0.00268***	-0.00349**
	(0.00073)	(0.00128)
Other psycholeptics (log(€))	0.00006	-0.0001
	(0.00035)	(0.00057)
No antidepressants	0.0019*	-0.0019
	(0.00083)	(0.0017)
Antidepressants (log(€))	0.00335***	0.0019**
	(0.00032)	(0.00066)
No other drugs	0.01107***	0.01426***
	(0.00022)	(0.00041)
Other drugs (log(€))	0.00086***	0.00175***
	(0.00007)	(0.00012)
Doctor consultations		
GP	-0.0064***	-0.00643***
	(0.00003)	(0.00006)
Psychiatrist	0.00049***	0.00085***
	(0.00008)	(0.00016)
Other specialists	-0.00152***	-0.00135***
	(0.00006)	(0.00013)
Absence from work		
Compensated days off work	-0.00026***	-0.00035***
	(0)	(0)
Hospitalization days	-0.00008***	-0.00012***
	(0.00001)	(0.00002)
Fixed effects		
Individual	Yes	Yes
Time	Yes	Yes
R^2	0.088343	0.089884
**Observations**	**312,904**	**98,615**

Field: Population having had a single WA from 2017 to 2019, resulting in at least a 4-day work stoppage, and for whom the information is available (N = 411,519). Note: * $p < 0.05$, ** $p < 0.01$, *** $p < 0.001$. Standard errors in parentheses. Interpretation: For people with a permanent contract at the time of WA, BZD overuse (compared to no BZD use, calculated for months t-4 to t-1) is associated with a 0.302 pp increase in WA probability at month t.
