# Supplementary material for: Impact of benzodiazepine use on the risk of occupational accidents
Source: PLoS One. 2024 Apr 16;19(4):e0302205. doi: 10.1371/journal.pone.0302205 (PMC11020385; doi:10.1371/journal.pone.0302205)
Supplement: S6 Table — Field: 10% random sample (N = 254,310). Note: * p < 0.05, ** p < 0.01, *** p < 0.001. Standard errors in parentheses. Interpretation: BZD overuse (compared to no BZD use, calculated for months t-4 to t-1) is not significantly (at a 5% threshold) associated with WA probability at month t. (PDF) [file pone.0302205.s007.pdf]

## S6 Table. Logit regression of WA risk in a population sample.

| | Coefficient (SE) | Odd ratios (95% Confidence Limits) |
|---|---|---|
| *BZDs (ref. no use)* | | |
| Overuse | -0.0026 | 1.009 |
| | (0.024) | (0.944-1.078) |
| Recent use | -0.0397** | 0.972 |
| | (0.0126) | (0.941-1.004) |
| Past use | 0.0535*** | 1.067 |
| | (0.0118) | (1.041-1.094) |
| *Chronic conditions* | | |
| Psychiatric | -0.3264*** | 0.521 |
| | (0.0327) | (0.458-0.592) |
| Other diseases | -0.1911*** | 0.682 |
| | (0.0168) | (0.639-0.729) |
| *Drugs reimbursed* | | |
| No other psycholeptics | -0.045* | 0.914 |
| | (0.0182) | (0.851-0.982) |
| Other psycholeptics (log(€)) | -0.033 | 0.968 |
| | (0.0178) | (0.934-1.002) |
| No antidepressants | 0.0143 | 1.029 |
| | (0.0232) | (0.940-1.127) |
| Antidepressants (log(€)) | 0.0373* | 1.038 |
| | (0.0179) | (1.002-1.075) |
| No other drugs | 0.0453*** | 1.095 |
| | (0.00512) | (1.073-1.117) |
| Other drugs (log(€)) | 0.0276*** | 1.028 |
| | (0.00322) | (1.022-1.034) |
| *Doctor consultations* | | |
| GP | -0.0922*** | 0.912 |
| | (0.00176) | (0.909-0.915) |
| Psychiatrist | 0.00028 | 1.000 |
| | (0.00591) | (0.989-1.012) |
| Other specialists | -0.0724*** | 0.930 |
| | (0.00385) | (0.923-0.937) |
| *Absence from work* | | |
| Compensated days off work | -0.0228*** | 0.977 |
| | (0.00019) | (0.977-0.978) |
| Hospitalization days | -0.0208*** | 0.979 |
| | (0.00161) | (0.976-0.982) |
| *Fixed effects* | | |
| Individual | Yes | |
| Time | Yes | |
| **Observations** | **254,310** | |

Field: 10% random sample (N=254,310). Note: * $p < 0.05$, ** $p < 0.01$, *** $p < 0.001$. Standard errors in parentheses. Interpretation: BZD overuse (compared to no BZD use, calculated for months t-4 to t-1) is not significantly (at a 5% threshold) associated with WA probability at month t.
