# Supplementary material for: Impact of benzodiazepine use on the risk of occupational accidents
Source: PLoS One. 2024 Apr 16;19(4):e0302205. doi: 10.1371/journal.pone.0302205 (PMC11020385; doi:10.1371/journal.pone.0302205)
Supplement: Attachment — Submitted filename: 2023-11-29 Response to reviewers.docx [file pone.0302205.s008.docx]

**I thank the reviewers for their analysis of the paper and very helpful comments. I describe below how I revised the manuscript in order to take systematically into account each of the reviewer’s comments.**

**Additional Editor Comments:**

The research question addressed by the author is very relevant. The methodology is sound but the author is required to make several amendments especially to the structure. Please do not use « I » but rather replace this pronoun by « the author ». Please move anything that includes results to the results’ section and anything that includes interpretation of results to the discussion section.
please provide as well a point-by-point response to both reviewers.

*Thank you for your comment. I have modified the manuscript to never use the first person (I use passive voice or “the author” instead). I have reorganized the results and discussion sections to meet your request.*

*Below is the point-by-point response to the reviewers.*

**Reviewers' comments:**

**Reviewer #1:** This study adds to the knowledge base by demonstrating the increased risks of work accidents associated with the intensity of benzodiazepine use.

Abstract
“we see an increase in risk in the month following the treatment discontinuation, which could be due to rebounding and catch-up effects.” Can the author specify how much the risk increased?

*I have added the value of the increase (+3.6%) in the abstract.*

The author mixed the pronouns “I” and “we” throughout the manuscript.

*I have replaced the first person with the passive voice throughout the manuscript. I use “we” only to comment on observations that include the reader.*

Introduction
(lines 109-117) The review of previous studies seems lengthy. I suggest that these paragraphs be shortened.

*I have reassembled the section on the limitations of existing studies, shortened the section on the presentation of results and merged the two paragraphs.*

(lines 118-125) The description of methodology and results should be removed or move to appropriate sections.

(lines 127-129) This sentence seems unnecessary
*I have deleted the corresponding sentences.*

Materials and methods
This section is written in sufficient detail.
(Line 244) cite the reference (source: Institute of Statistics, INSEE) properly.

*I have added the references to the bibliography.*

(line 244-245) “..refers to the double selection of people who are employed and have had an accident at work.” This is a confusing statement. Please rephrase it.

*I have reworded the sentence: “…and refers to a double selection effect: people with a WA are employed and more likely to work in hazardous jobs”.*

(lines 249-250, line 255) Please present the results for the percentage of people who experienced WA in Table 1 in terms of % as you did in the text (39%, 40%, 39%).

(lines 252-254) “Accidents at the workplace constitute the majority of WAs (gathering workplace and commuting accidents) and affected 87% of the population experiencing a WA in 2017, compared to commuting accidents affecting 14% of the same population. Please present the results in a Table as well as in the text.

*I have slightly modified the first part of the statistics section:*

- *I have added age distribution figure as supplementary material.*
- *I have modified Table 1 to focus on accident statistics. It contains the number and proportion of people experiencing the different type of accidents, as well as in the text.*

The subsection, Statistics, should fall under the results section.

*Modification made.*

Results
(line 291) explain the acronym (like pp) at its first appearance.

*Modification made.*

(lines 298-300) This reflects the health deterioration, which probably leads to a decrease in the probability of being exposed (loss of job, decrease in working time, and decrease in exposure. This is an interpretation of the results, which should better be placed in the discussion section.
There are also some other statements in the results section that should instead belong in the discussion.

*I have moved all the sentences interpreting the results to the discussion section.*

Discussion
Please add suggestions for future studies.

*I have added a paragraph at the end of the discussion.*

**Reviewer #2:** L21: avoid using first person throughout the manuscript i.e., "me" and "I". Avoid changing from past to present tense throughout the manuscript

*I have changed the use of first person to passive voice throughout the manuscript. I have also modified manuscript to consistently use the present tense, except when I am commenting on something in the past (for example, “The average age of the study population was 37 in January 2017”).*

L23: shows a decreased risk of WA for short-term BZD use compared to what? Please specify this in the abstract.

*I have changed the sentence: “The results show a reduction in the risk of WA after a short period of BZD use (one month) compared with no use at all”.*

L25: Please explain in the abstract what a "percent increase" of BZD use means. Is it a percent increase in the dose (e.g., mgs)? This should be clear.

*I have changed the sentence: “The intensity of use results in a greater risk of WAs: a 1% increase in BZD use (expressed as the amount reimbursed)”.*

L28-30: you state here that there is a WA risk induced by BZDs at the beginning of treatment; however, you state in L25: that there is a decreased risk up to one month. Please clarify which is correct.

*There is no contradiction between the two sentences, because one refers to the direct effect of BZDs and the other to what was observed in the study, which may also be the consequence of adaptation and risk reduction measures. The adverse effects of BZDs are likely to increase the risk of WAs, and the doctor's preventive action may be concentrated at the start of treatment and become less pronounced over time. This study shows a reduction of the risk during the month after starting to use BZDs, not necessarily from a direct effect of the treatment but rather from prevention and risk reduction. The message I would like to convey with this sentence is that preventive action against WAs is required during all the BZD treatment and even after the treatment has been discontinued, and not that doctors should stop to prevent risk when treatment starts.*

*I have changed the sentence to make it clearer: “Health professionals and BZD users should be made aware of the WA risk induced by the use of BZDs, particularly after prolonged use and after discontinuation of treatment.”*

L59: BZDs are also used in seizure prophylaxis and acute management. Sometimes long-term use of BZDs is required (e.g., clonazepam) and this should be addressed.

*In France, BZDs are only indicated for short-term use (see French National Authority for Health guidelines cited in the article). The only indication for clonazepam is the treatment of epileptic seizure.*

*Reference for clonazepam indication:* [*https://www.vidal.fr/medicaments/substances/clonazepam-4157.html*](https://www.vidal.fr/medicaments/substances/clonazepam-4157.html)

L62: You should state the effects of BZD use e.g., sedation etc.

*I have changed the sentence: “The effects of BZDs may lead to a higher risk of WAs, as they have sedative and cognitive effects”. I do not go into details about these adverse effects, as I am more precise two paragraphs later.*

L437: You need to define what you mean by hypnotic BZDs and anxiolytic.

*I have added a definition of the terms hypnotic and anxiolytic BZDs in the third paragraph of the introduction, along with recommended treatment durations:*

*“One particular class of drugs deserves our attention: the benzodiazepines (BZDs), which are used broadly as anxiolytics and hypnotics. Short-acting BZDs are used as sleeping pills (hypnotic BZDs), while long-acting BZDs are used as anxiolytics (anxiolytic BZDs). As the risk of dependence increases with the duration of treatment, the French National Authority for Health (HAS) recommends limiting the treatment duration to four weeks for hypnotic BZDs and twelve weeks for anxiolytic BZDs (9,16,17).”*

L441: I am not sure intensity of use is the correct term. Is this referring to dose or duration?

*I use the log of amount reimbursed as a proxy for the dose. This method is described in the “Econometric strategy” section: the log of the amount reimbursed for BZDs over the last four months (+1), which expresses the intensity of treatment”.*

L503: You should include BZD withdrawal here.

*I have added it in the conclusion: “Treatment discontinuation is associated with an increased risk of WAs that may arise from rebound effects, withdrawal symptoms, and catch-up effects.”*

L511: This recommendation should only apply to specific indications for BZDs. Long-term use can be appropriate.

*In France, there is no recommendation for the use of BZDs after twelve weeks. Long-term prescribing only exists outside the scope of marketing authorization. The literature cited highlights the risk of adverse effects when treatment is prolonged. See the recommendations here (in French, with abstract in English): Revet A, Yrondi A, Montastruc F. Règles de bon usage des benzodiazépines. Presse Médicale. 2018 Oct 1;47(10):872–7.*

Additional Comments:
1) You should be careful using the term "overused" when referring to BZD use as there are indications where this is necessary. It is, however, longer that the recommended use for sleep or mild-moderate anxiety. The introduction should outline the guidelines for BZDs.

*I have added guidelines for BZDs in the introduction. As there is no indication in France for use beyond twelve weeks, I consider longer use to be overuse, and I indicate it in the “Econometric strategy” section: “BZD overuse corresponds to exceeding the treatment duration recommended by the HAS for anxiolytic BZDs: 12 weeks at most”.*

2) The post-treatment period could be significantly affected by intermittent BZD use. People can typically hold onto, for example, 50 diazepam, for a long period of time and still be using it.

*I have added this in the limitation section: “The post-treatment period could also be affected by intermittent use, particularly in the case sales in large packages.”*

3) There seems to be key statistical values missing in the results text (e.g., confidence intervals). This needs to be included for all results; results should not just be contained in tables.

*As I provide the dispersion measures in the form of standard errors, I have added significance through the p-value in the text, for all results. For the sake of readability, I have indicated standard errors only in the tables.*
