# Supplementary material for: Impact of benzodiazepine use on the risk of occupational accidents
Source: PLoS One. 2024 Apr 16;19(4):e0302205. doi: 10.1371/journal.pone.0302205 (PMC11020385; doi:10.1371/journal.pone.0302205)
Supplement: Attachment — Submitted filename: 2024-03-11 Response to reviewer.docx [file pone.0302205.s009.docx]

**I thank the reviewer for his/her analysis of the paper and his/her very helpful comments. I describe below how I revised the manuscript in order to take systematically into account each of the reviewer’s comments.**

Journal Requirements:

Please review your reference list to ensure that it is complete and correct. If you have cited papers that have been retracted, please include the rationale for doing so in the manuscript text, or remove these references and replace them with relevant current references. Any changes to the reference list should be mentioned in the rebuttal letter that accompanies your revised manuscript. If you need to cite a retracted article, indicate the article’s retracted status in the References list and also include a citation and full reference for the retraction notice.

**Although I use bibliography management software, I have no information about a retracted reference in the bibliography. The only change I have made is to reference 25, because the article has been published.**

Reviewer #3: The manuscript could be further improved.

Line 21, the complete name of the two-way fixed effects test is to be provided.

**“Two-way FE” refers to the econometric approach used in this study rather than to a one- or two-sided test of statistical significance. I have modified the sentence to show the name of the model used: “A linear probability model with two-way fixed effects is used.”**

Line 116-125, whether the database is an open-source type and information on the type of data/file format that could be extracted and accessed is to be mentioned. Source link to be provided (if any).

**The database is not open-source. Publicly sharing SNDS data is prohibited by law, according to the French national data protection agency (Commission Nationale de l’Informatique et des Libertés, CNIL). Researchers can request access from the National Health Data Hub (**[**www.health-datahub.fr**](http://www.health-datahub.fr)**). I have provided this information in the form sent to the editor (“Data availability” section).**

Line 131 -143 is to be placed before section (Line 115).

**It seems more logical to me to place the "population" section after the "data" section, given that the choice of population selection is based on the source of the data and that this paragraph refers to the previous one. Furthermore, looking at recent publications in PLOS ONE, it appears that the "data" section is placed before the "population" section in the articles consulted (see for example** [**https://journals.plos.org/plosone/article?id=10.1371/journal.pone.0298068#sec002**](https://journals.plos.org/plosone/article?id=10.1371/journal.pone.0298068#sec002) **or** [**https://journals.plos.org/plosone/article?id=10.1371%2Fjournal.pone.0298516#sec006**](https://journals.plos.org/plosone/article?id=10.1371%2Fjournal.pone.0298516#sec006)**)**

Line 146-147, the statement is unclear and requires revision.

**I have replaced the sentence with the following: “The model used for estimation is a linear probability model. The dependent variable is the monthly WA probability. The independent variables are related to healthcare utilization in the previous four months; the first four periods are therefore not used for estimation.”**

Line 218, while fixed effects can help control for time-constant heterogeneity, they are unable to fully address variables that are completely missing from the dataset.

**Month*year fixed effects control seasonal/temporal variables that are independent of individual characteristics. Individual fixed effects control variables that are linked to individuals but do not vary over time. These fixed effects cannot handle unobserved variables that vary over time and between individuals. If these variables are linked to both individual characteristics and the outcome, they can be a source of bias.**

**I have added this in the “Econometric strategy” section:**

**Line 148-150: “Individual and monthly fixed effects are used in the model to control, respectively, variables linked to individuals but which do not vary over time and seasonal/temporal variables independent of individual characteristics.”**

**Line 216-219: “Two-way fixed effects are especially helpful where individual social and economic data are lacking. Individual fixed effects can control for certain unobserved variables (such as social origin). However, fixed effects cannot deal with unobserved variables that vary over time and between individuals.”**

Line 220-222, although this approach can offer valuable insights and help address the missing data, it may not fully address potential sources of bias or confounding related to missing variables. What about the social origin and professional exposure variables (Line 217-218)? More information is to be provided/presented/discussed.

**I consider that social origin is fixed over time and is therefore eliminated by individual fixed effects. Job type can vary with individual characteristics and over time, and be source of bias. This is why I carry out a robustness check on two sub-populations: with and without long-term contracts (see section ‘robustness checks’). As far as occupational exposure is concerned, I consider two possibilities for the variable to affect the result:**

- **If there is a change in occupation exposure because of BZD use, I consider it part of the observed effect. Thus, in the ‘discussion of results’ section, I interpret the results in terms of medical effects and occupational exposure.**
- **If there is an omitted variable that affect both the probability of using BZDs and occupational exposure, it may bias the results.**

**To clarify these points, I have added a paragraph in the ‘limitations’ section.**

Line 225-231, more baseline characteristics are to be provided and presented in table form.

**The only demographic variables available for the entire population in the database are age and sex. The other variables relate to health care consumption. I provide descriptive statistics for discrete variables in Table 2 and for continuous variables in Table 3. Age and sex statistics are provided off-form because they are the only ones not related to care consumption. In addition, I provide the age distribution as supporting information. Unfortunately, the database does not contain socio-economics variables for the entire population.**

Table 2, 0 & 1 are to be denoted. Frequency is to be provided apart from %.

**I have added two frequency columns corresponding to %.**

Table 3, Q3, and P90 are to be denoted.

**I have added the following in the legend: “Q3 is the third quartile of the distribution and P90 is the 90th percentile.”**

Table 4, the word 'stepwise' and Line 290 'the variable is not used in this specification' is not clear. More information is to be provided in the table footnote to describe the models. Model fit indices is to be presented.

**I have replaced the word ‘stepwise’ with ‘variable added progressively’ in the text and in the table title. I renamed the column, so that the text, columns and footnote refer to the different specifications.**

**I added R^2^ to the bottom of the table.**

Table 5, the selection method is to be stated.

**In this study, the word ‘stepwise’ did not refer to a variable selection method, but to the fact that I added variables gradually. I rephrase without the word ‘stepwise’, because there is no selection in the regression presented in Table 5.**

Line 282-283, the sentence requires improvement.

**I have improved the sentence: ‘Medical consultations are negatively associated with the WA risk (except for psychiatrists, whose effect is not significant): an additional GP consultation in the last four months reduces the probability of WA by 0.27 pp (8.3%, p<0.001) (by 0.19 pp, 5.8%, p<0.001 for consultations with other specialists).’**

Decimal point for percentage figures is to be standardized.

**Modification made.**

S6 Table & S6 Table Page 31, 10th is to be denoted.

**I changed the title and footnote of the table: ‘S6 Table. Logit regression of WA risk in a population sample. Field: 10% random sample (N=254,310).’**
