# Supplementary material for: Impact of benzodiazepine use on the risk of occupational accidents
Source: PLoS One. 2024 Apr 16;19(4):e0302205. doi: 10.1371/journal.pone.0302205 (PMC11020385; doi:10.1371/journal.pone.0302205)
Supplement: Attachment — Submitted filename: 2024-03-25 Response to reviewers.docx [file pone.0302205.s010.docx]

Response to reviewers

25/03/2024

Thank you for responding to the statistical reviewer’s comments. One final concern is the way results are reported. The author should keep in mind that this is not a thesis on statistics. We are aware that statistical analyses were performed to provide the readers with meaningful results.

1-Please avoid using the statistical technical terms in the tables. You defined your dummy variables in the methods. In the results’ sections give them a descriptive title: «Percentage of Work accidents and benzodiazepine use? »

**I have changed the title to “Percentage of work accidents and benzodiazepine use”.**

2-Please provide at the beginning of the results in the section that is called « statistics »once more the number of the population studied and mean and SD of age. Average should not be used in scientific reporting.The title of this section can be improved as well. We know that the author is reporting « statistics » what we would like to see is « what are the results about?

**I have added sample size and SD of age. I changed the section title to: “Work accidents and healthcare use in the population”.**

3-Please provide R2 in Table 5 as suggested by the statistical reviewer.

**I have added R2 in the table.**

4-Please put descriptions instead of numbers in tables 4 and 5. The reader is not supposed to guess what is (1) (2)…et. Anything that is not clear in the tables should be defined in the footnote.

**I have changed to “Specification (1)”, as in the text and in the footnote.**

5-Please remove No=1 or Yes=1 or anything similar from the columns in Tables. The author is asked to rephrase the variable in a way that reflects what he aims to. For instance: Male sex is enough to understand that it is(1). The author can put Ref. Female (to point out that the reference is 0).

**I have made modifications to Tables 4 and 5.**

**I would like to thank reviewers and editor again for their valuable advice. I hope that the manuscript is now ready for publication in Plos One. Of course, I remain at your disposal to make any changes you may wish.**
